# Proteomic Analysis of Outer Membrane Proteins from *Salmonella* Enteritidis Strains with Different Sensitivity to Human Serum

**DOI:** 10.1371/journal.pone.0164069

**Published:** 2016-10-03

**Authors:** Bartłomiej Dudek, Eva Krzyżewska, Katarzyna Kapczyńska, Jacek Rybka, Aleksandra Pawlak, Kamila Korzekwa, Elżbieta Klausa, Gabriela Bugla-Płoskońska

**Affiliations:** 1 Department of Microbiology, Institute of Genetics and Microbiology, Faculty of Biological Sciences, University of Wroclaw, Wroclaw, Poland; 2 Department of Immunology of Infectious Diseases, Hirszfeld Institute of Immunology and Experimental Therapy, Polish Academy of Sciences, Wroclaw, Poland; 3 Dialab Laboratory, Wroclaw, Poland; 4 Regional Centre of Transfusion Medicine and Blood Bank, Wroclaw, Poland; Robert Koch Institut, GERMANY

## Abstract

Differential analysis of outer membrane composition of *S*. Enteritidis strains, resistant to 50% normal human serum (NHS) was performed in order to find factors influencing the resistance to higher concentrations of NHS. Ten *S*. Enteritidis clinical strains, resistant to 50% NHS, all producing very long lipopolysaccharide, were subjected to the challenge of 75% NHS. Five extreme strains: two resistant and three sensitive to 75% NHS, were chosen for the further analysis of outer membrane proteins composition. Substantial differences were found in the levels of particular outer membrane proteins between resistant and sensitive strains, i.e. outer membrane protease E (PgtE) was present mainly in resistant strains, while sensitive strains possessed a high level of flagellar hook-associated protein 2 (FliD) and significantly higher levels of outer membrane protein A (OmpA).

## Introduction

For many years bacteria from the genus of *Salmonella* have remained the leading cause of gastrointestinal diseases, posing a significant epidemiological threat to the community. Every year 94 million infections, caused by nontyphoidal *Salmonella* (NTS) organisms, are recorded worldwide [1]. Moreover, these epidemiological data are probably highly underestimated [2], as most salmonellosis cases are not reported due to the short course of the disease and no hospitalization. It is estimated that in the USA only 1–5% of the cases are laboratory confirmed and reported to the Centers for Disease Control and Prevention (CDC) [3]. In addition to the typical course of salmonellosis, an infection with *Salmonella* can often lead to a parenteral form and eventually to sepsis. *Salmonella enterica* subsp. *enterica* Enteritidis (*S*. Enteritidis) and S*almonella enterica* subsp. *enterica* Typhimurium (*S*. Typhimurium) are the two most common clinical serovars (serological variants) isolated worldwide as causative agents of salmonellosis [1]. In all regions of the world except North America and Oceania, serovar *S*. Enteritidis is more widespread than *S*. Typhimurium (65% and 12% of all isolates worldwide, respectively) [4,5].

In 2012 nearly 77% of the total cases of human salmonellosis was caused by *S*. Enteritidis—currently the most commonly isolated serovar in Poland [6]. *S*. Enteritidis—a facultative intracellular pathogen—is the prominent reason responsible for acute gastroenteritis because it is widespread in poultry products and it is highly resistant to harsh and adverse conditions.

Complement resistance is a crucial factor in the development of a systemic infection and bacteremia for many pathogenic bacteria. The complement system is composed from a combination of plasma proteins. The enzymatic cascade activation causes opsonization of the invading microbe and a release of chemotactic compounds that promote leukocyte recruitment and phagocytosis of invading bacteria. In addition, formation of the terminal complement complex leads the lysis of the cell membrane. Three different pathways: classical, alternative and lectin pathway, can activate the complement system. Three homologous glycoproteins: C3, C4 and C5, play the main role in complement activation and are responsible for the interaction with other complement components and precursors for proteins promoting the activation of the complement cascade.

Due to their high virulence and a number of features that predispose *Salmonella* to survive in the serum, it is necessary to constantly investigate and explore the structural elements of the bacteria, which are responsible for the resistance to the immune response of the host. An important virulence element is the ability to change the structures of the outer envelopes, which enables a survival and multiplication of bacteria in an extremely adverse environment. *Salmonella* resistance to the bactericidal activity of serum is determined by several factors, like variable lipopolysaccharide (LPS) O-specific chain length, polysaccharide capsules, specific outer membrane proteins (OMPs) or the presence of fimbriae on the cell surface.

The unique chemical structure of LPS is responsible for several specific properties of bacterial cells. LPS consists of three structural domains: lipid A, core oligosaccharide and the O-specific antigen (O-antigen). The O-specific antigen is a polysaccharide, made up from repeating oligosaccharide subunits [7]. It is the distal part of LPS exposed to the external environment and responsible for the serological properties of bacteria, with great chemical diversity among bacterial strains. LPS forms a durable barrier around a bacterial cell, to mask and protect it against the host immune system [8]. The LPS O-specific chain plays an important role in the generation of serum complement resistance of various Gram-negative bacteria: *S*. Typhimurium [9], *Yersinia enterocolitica* [10] or *Klebsiella pneumoniae* [11]. Lipopolysaccharide molecules from some of the *Salmonella* strains exhibit the so-called modal length distribution. That type of distribution is characterized by the occurrence of three clearly distinct LPS fractions, differing in the number of repeating subunits in the O-specific antigen: low molecular weight LPS (LMW-OAg) with 15 repeating subunits, long (L-OAg) with 16–35 repeating subunits and very long (VL-OAg) comprising more than 100 repeating subunits [12]. The lipopolysaccharide molecules of *S*. Typhimurium and *S*. Enteritidis possess modal length distribution, with *wzz* gene controlling the synthesis of L-OAg and *wzz*_*fepE*_ gene responsible for the expression of the VL-OAg [9], while *S*. Typhi produces LPS without any apparent modal distribution [13]. The long and very long O-specific chains of LPS hamper efficient attachment of complement membrane attack complex (MAC) to the lipid part of the outer membrane, thereby preventing a bacterial cell from lysis. Crawford et al. [14] also show the participation of VL-OAg LPS in the *Salmonella* survival in bile, which increases the pathogenicity of *S*. Typhimurium strains. In addition, Bravo et al. report that strains of *S*. Enteritidis and *S*. Typhimurium, with L-OAg LPS only, show lower resistance to serum than mutants with VL-OAg LPS only [12].

OMPs act in the cell as active factors against the negative influence of external environment, which can cause various levels of their expression. Due to the peripheral location in a bacterial cell, OMPs are active in the process of cell adaptation to fluctuating habitat conditions [15]. Moreover, they are thought to be a substantial component of the bacterial cell surface that is involved in the inhibition of complement activity and the activation of the complement system. Some OMPs amplify the sensitivity of Gram-negative bacteria to the bacteriolytic action of complement components [16–18]. Binding of OmpK36 from *K*. *pneumoniae* to C1q proteins of complement leads to activation of the complement classical pathway and the subsequent deposition of C3b and C5b-9 on the porin [19]. Among OMPs there is a number of proteins involved in *Salmonella* resistance to serum, including PagC, PgtE, Rck and TraT [20–23]. The mechanisms by which OMPs interact with the complement system have not been fully characterized and are still under investigation. It has been shown that the presence of PagC in the outer membrane of *S*. Choleraesuis results in serum resistance [20]. Studies of Riva et al. suggest that in *S*. Typhimurium PgtE mediates in the generation of serum resistance by its ability to degrade the complement components: C3, C3b, C4, C5, and C4b [22]. In turn, Ho et al. show that protein Rck contributes to the inhibition of MAC formation by binding to protein C9 and factor H of the complement system [21]. It has also been shown that protein TraT of *S*. Typhimurium and *S*. Enteritidis, can inhibit the formation of MAC by binding to factor 6, which is a part of the complement cascade system [23]. Bugla-Płoskońska et al. confirmed that there are certain OMPs characteristic for the serum-sensitive and serum-resistant phenotypes of *Salmonella* O48 strains [24]. A number of previous studies have investigated the OMPs profiles of different *Salmonella* strains [25–28], but only one study on outer membrane proteins of *S*. Enteritidis has been published [29] to date. As the data concerning virulence factors of *S*. Enteritidis is generally limited, the objective of the present investigation was a comparative analysis of the outer membrane composition of *S*. Enteritidis strains, to find a correlation between the expression of specific OMPs and the level of resistance to human serum. Ten strains, which were found to be resistant to normal human serum (NHS, 50%), were further tested for their susceptibility to the increased concentration of NHS (75%). After the analysis of their LPS molecules length distribution, 5 outlying strains, i.e. 2 extremely resistant and 3 extremely sensitive, were chosen for a differential analysis of their OMPs composition. Isolated OMPs were separated in 2D gel electrophoresis and selected spots were identified using mass spectrometry.

## Materials and Methods

### Bacterial strains

Ten clinical strains of *S*. Enteritidis were isolated from the faeces of patients with symptoms of diarrhea (Dialab Laboratory). *S*. Enteritidis were identified, using biochemical tests and serotyping with agglutination tests with specific O and H antisera, and classified according to the Kauffmann-White-Le Minora scheme [30]. The strains are available in the collection of the Department of Microbiology at the University of Wroclaw (Table 1).

**Table 1:** *Salmonella* Enteritidis strains from the collection of the Department of Microbiology, the University of Wroclaw.

No	Date of sampling [year/month]	Number of *S*. Enteritidis strain	Number of PCM* *S*. Enteritidis strain
1	2011/04	670	2813
2	2011/05	888	2808
3	2012/03	27	2817
4	2012/04	351	2814
5	2012/05	3686	2809
6	2012/08	851	2812
7	2012/08	920	2811
8	2012/09	1120	2815
9	2012/09	4574	2807
10	2012/11	1227	2816

* *S*. Enteritidis strains have been submitted to the Polish Collection of Microorganisms (PCM).

### Serum

Normal human serum (NHS) from a group of healthy adults, who had not received any antimicrobial drug treatment for 6 months, was obtained from Regional Centre of Transfusion Medicine and Blood Bank in Wroclaw. This was conducted by Elżbieta Klausa according to the principles expressed in the Declaration of Helsinki. Experiments were performed with NHS samples taken from individual donors and mixed together. Blood samples were taken into aseptic dry tubes or tubes with an anticoagulant. After collection, the samples were stored at room temperature for 1 h to allow separation and then centrifuged for 10 min at 1200g to pellet cells and platelets. The serum samples were frozen in 0.5 ml aliquots at -70°C and stored for no longer than 3 months. The required volume of serum was thawed immediately before use and each portion was used only once. For this study, only serum samples lacking hemolysis and with negative test results for HIV, HCV and *Treponema pallidum* antibodies, a negative HBs antigen test, and a negative viral genome screen (HIV RNA, HBV DNA, HCV RNA) were used.

### Serum C3 concentration

The C3 concentration in the mixed serum (NHS) was quantified by a radial immunodiffusion test (Human Complement C3 & C4 ‘Nl’ Bindarid Radial Immunodiffusion Kit; The Binding Site Group Ltd.).

### Serum susceptibility assay

Ten *S*. Enteritidis strains were subjected to the challenge of 50% NHS. All strains were found to be resistant to this concentration of NHS, so the strains were further tested for their susceptibility with an increased concentration of NHS (75%).

The bactericidal activity of NHS was determined as described previously [31] with slight modifications [32]. Bacterial strains were cultured overnight at 37°C in 3 ml of Lysogeny Broth Lennox (LB) (Sigma). After overnight incubation, bacterial cells (500μl) in the early exponential phase were transferred into 5 ml of fresh LB medium and incubated at 37°C for 1 h in a water bath with rotation at 200 rpm to achieve an OD_600_ of 0.3. Next, bacteria cells were collected by centrifugation (1500g for 20 min at 4°C). The pellet was resuspended in 3 ml of physiological saline (0.9% NaCl) (POCH) and then diluted in the same saline to produce a suspension of approximately 10^6^ cells/ml. Aliquots of the cell suspension were mixed with an appropriate volume of NHS and incubated at 37°C for 0, 60 and 180 min (T_0_, T_1_ and T_3_, respectively) in a water bath with rotation at 200 rpm. Appropriate dilutions were then spread in duplicate onto nutrient agar plates and incubated at 37°C for 24 h. The average number of colony-forming units per milliliter (CFU/ml) was calculated from the replicate plate counts. The survival rate for T_1_ and T_3_ was calculated as a percentage of the cell count for T_0_ (set at 100%). Strains with survival rates of >100% after 180-min incubation with serum were considered resistant, and those with survival rates of <100% were considered susceptible to the bactericidal action of NHS. Strains with survival rates between 50% and 100% were considered to have intermediate (moderate) resistance to NHS. NHS decomplemented by heating at 56°C for 30 min (heat inactivated serum, HIS) was used as a control [33]. Each test was performed three times.

### Lipopolysaccharide isolation and analysis by SDS-PAGE

Extraction of LPS was performed using a commercial RNA isolating reagent: Tri-Reagent (Sigma-Aldrich) according to Yi and Hackett [34]. 10 mg of lyophilized bacterial cells were suspended in 200 μl of Tri-Reagent and the cell suspension was then incubated at room temperature for 10 min for complete cell homogenization. After incubation, 200 μl of chloroform was added to create a phase separation. The mixture was then vigorously vortexed and incubated at room temperature for additional 10 min. The resulting mixture was centrifuged at 12 000g (Sigma, Heraeus Fresco 21) for 10 min to separate the aqueous and organic phase. The aqueous phase was transferred to a new 1.5 ml centrifuge tube. Distilled water (100 μl) was added to the organic phase. The mixture was vortexed, incubated at room temperature for 10 min, and centrifuged at 12 000g for 10 min. The upper aqueous phases from both steps were combined. Two additional water extraction steps were repeated. The combined aqueous phase was lyophilized. After lyophilization we used the cold magnesium precipitation procedure according to Darveau and Hancock for the purification of LPS [35]. LPS was dissolved in 500 μl of 0.375 M magnesium chloride (POCH) in 95% ethanol, stored at -20°C, followed by centrifugation at 12 000g for 15 min. The pellet was suspended in 200 μl of distilled water and lyophilized. LPS extracts were analyzed by discontinuous SDS-PAGE using Laemmli buffer system [36]. Samples were applied to the slabs after mixing with Laemmli buffer and heating at 98^○^C for 7 minutes. Gel electrophoresis was performed using 6% polyacrylamide stacking gel and 15% separating gel. The SDS-PAGE separation of LPS was performed at constant voltage (120V), for 90 minutes using a Mini-Protean Tetra Cell apparatus (Bio-Rad). The separated LPS was visualized using silver staining according to Tsai and Frasch [37] with Fomsgaard [38] and photographed under white light using a GelDoc XR imaging system (Bio-Rad).

### OMPs isolation and preparation

The isolation of OMPs was performed according to Murphy and Bartos [39] with own minor modifications [24]. Bacterial strains were cultured overnight at 37°C in 25 ml LB medium. After growth, the cells from the overnight culture were harvested (1500g at 4°C for 20 min) and suspended in 1.25 ml 1 M sodium acetate (POCH), 1mM β-mercaptoethanol (Merck). Subsequently, 11.25 ml water solution containing 5% (w/v) Zwittergent Z 3–14 (Calbiochem) and 0.5 M CaCl_2_ (POCH) was added. This mixture was stirred at room temperature for 1 h. To precipitate nucleic acids, 3.13 ml of 96% (v/v) cold ethanol (POCH) was added very slowly. The mixture was then centrifuged at 17000g at 4°C for 10 min. The proteins were precipitated from the supernatant by the addition of 46.75 ml of 96% (v/v) cold ethanol and centrifuged at 17000 g at 4°C for 20 min. The pellet was left to dry at ambient temperature and then suspended in 1.5 ml 50 mM Trizma-Base (Sigma-Aldrich) buffer, pH 8.0 containing 0.05% (w/v) Zwittergent Z 3–14 and 10 mM EDTA (Sigma-Aldrich) and stirred at room temperature for 1 h. The solution was kept at 4°C overnight. Insoluble material was removed by centrifugation at 12 000g at 4°C for 10 min, with OMPs present in the supernatant.

Total protein concentration was measured using a commercial BCA Protein Assay Kit (Thermo Scientific).

### Two dimensional gel electrophoresis

The OMPs were separated with 4–7 pH immobilized gradient IPG strips (7 cm) (Bio-Rad). 2-DE was carried out with the Mini-PROTEAN Tetra Cell System (Bio-Rad). Isoelectric focusing (IEF) was conducted by stepwise increase of voltage as follows: 250 V, 20 min; 4000 V, 120 min (linear) and 4000 V (rapid), until the total volt-hours reached 14 kVh. IPG strips were then loaded onto the top of 1-mm slabs comprised of a 9% polyacrylamide stacking gel and 12,5% polyacrylamide separating gel, using 0.5% agarose (Bio-Rad) in the running buffer. Electrophoresis was performed at 4°C with constant power current (1 W) until the dye front reached the bottom [40–42]. The protein spots were visualized by the silver staining method [43] and in gels used for mass-spectrometric identification of proteins – by Coomassie Brilliant Blue (Bio-Rad). Band patterns were visualized under white light and photographed using ChemiDoc MP system (Bio-Rad). Image spots of proteins were analyzed by PDQuest software (Bio-Rad).

### In-Gel Protein Digestion and MS Protein Identification

All solvents used for digestion, MS preparation and analysis were of LC-MS grade and purchased from Merck Millipore. Ammonium bicarbonate eluent additive for LC-MS, dithiothreitol and iodoacetamide were from Sigma. Sequencing Grade Modified Trypsin was obtained from Promega.

After isolation, 2-DE separation and staining with Coomassie Brilliant Blue method protein spots of interest were excised from the gel and subjected to the in-gel tryptic digestion according to the method described by Shevchenko et al. [44]. Briefly, after destaining (100 mM NH_4_HCO_3_/acetonitrile, 1:1, vol:vol), reduction (10 mM dithiothreitol in 100 mM NH_4_HCO_3_) and alkylation (55 mM iodoacetamide in 100 mM NH_4_HCO_3_), a suitable volume of 13 ng/μl trypsin in 10 mM ammonium bicarbonate containing 10% (vol/vol) acetonitrile was added to the excised gel spot cut into cubes. The obtained peptides were extracted from the gel, concentrated and desalted with the Pierce C18 tip, and subjected to mass spectrometry analysis using the MALDI TOF ultrafleXtreme instrument (Bruker Daltonics). 10 mg/ml of α-cyano-4-hydroxycinnamic acid (Bruker) in acetonitrile/0.1% TFA in H_2_O (1:1, vol:vol) was used as the eluent of peptides from the Pierce C18 tip directly on a MALDI plate. Spectra were acquired in positive reflector mode, averaging 2000 laser shots per MALDI-TOF spectrum. OMPs identification was achieved using a bioinformatics platform (ProteinScape, Bruker Daltonics) and MASCOT (Matrix Science) as a search engine to search protein sequence databases (NCBI, SwissProt) using the peptide mass fingerprinting method.

## Results

### Bactericidal Assay of NHS

As C3 is a crucial protein in the activation of the complement cascade, standard analysis of C3 protein levels in NHS used for experiments were performed. C3 concentration was 1330 mg/L, which is in the range of standard values (970–1576 mg/l for males and 1032–1495 for females).

All the ten strains of *S*. Enteritidis were found to be resistant to NHS in the standard test of the bactericidal activity with 50% NHS (Table 2).

**Table 2:** Bactericidal activity of 50% NHS and 50% HIS decomplemented by heating at 56°C for 30 min against *S*. Enteritidis strains.

Bactericidal activity of 50% NHS^1^							Bactericidal activity of 50% HIS^2^		
Strain	CFU/ml^3^			Survival rate of cells at T_1_ [%]	Survival rate of cells at T_3_ [%]	RP^4^	CFU/ml		Survival rate of cells at T_3_ [%]
	T_0_	T_1_	T_3_				T_0_	T_3_	
27	2.36 x 10^6^ ± 2.14 x 10^5^	3.71 x 10^6^ ± 8.52 x 10^5^	9.07 x 10^6^ ± 2.54 x 10^6^	155.8 ± 23.2	379.9 ± 77.7	R	1.83 x 10^6^ ± 2.32 x 10^5^	1.67 x 10^8^ ± 3.00 x 10^7^	9092.2 ± 699.3
851	1.80 x 10^6^ ± 1.05 x 10^5^	3.28 x 10^6^ ± 4.51 x 10^5^	6.96 x 10^6^ ± 6.91 x 10^5^	183.2 ± 33.0	387.6 ± 48.8	R	2.74 x 10^6^ ± 4.31 x 10^5^	1.25 x 10^8^ ± 4.26 x 10^7^	4462.9 ± 851.2
670	2.40 x 10^6^ ± 1.46 x 10^5^	2.96 x 10^6^ ± 3.30 x 10^5^	4.84 x 10^6^ ± 2.27 x 10^6^	123.3 ± 7.3	202.3 ± 97	R	2.30 x 10^6^ ± 1.36 x 10^5^	3.77 x 10^7^ ± 1.16 x 10^7^	1624.9 ± 440.1
888	2.14 x 10^6^ ± 1.75 x 10^5^	2.07 x 10^6^ ± 3.75 x 10^5^	2.35 x 10^6^ ± 3.27 x 10^5^	96.2 ± 10.9	109.3 ± 6.6	R	3.06 x 10^6^ ± 6.81 x 10^4^	5.55 x 10^7^ ± 3.80 x 10^6^	1816.1 ± 99.8
1120	2.51 x 10^6^ ± 2.80 x 10^5^	2.43 x 10^6^ ± 3.37 x 10^5^	2.55 x 10^6^ ± 3.14 x 10^5^	96.5 ± 4.6	101.4± 2.1	R	1.60 x 10^6^ ± 9.85 x 10^4^	1.79 x 10^7^ ± 3.67 x 10^6^	1112.6 ± 174.2
351	3.15 x 10^6^ ± 3.17 x 10^5^	3.71 x 10^6^ ± 2.76 x 10^5^	6.77 x 10^6^ ± 1.66 x 10^5^	118.3 ± 10.0	216.3 ± 23.1	R	3.31 x 10^6^ ± 2.88 x 10^5^	3.36 x 10^7^ ± 7.61 x 10^6^	1016.7 ± 208.0
920	2.06 x 10^6^ ± 7.51 x 10^4^	2.53 x 10^6^ ± 2.01 x 10^5^	3.60 x 10^6^ ± 5.11 x 10^5^	123.0 ± 8.7	174.7 ± 22.1	R	1.92 x 10^6^ ± 2.80 x 10^5^	3.67 x 10^7^ ±1.19 x 10^7^	1885.3 ± 421.5
1227	1.54 x 10^6^ ± 8.33 x 10^4^	1.89 x 10^6^ ± 1.54 x 10^5^	2.57 x 10^6^ ± 2.20 x 10^5^	122.4 ± 5.5	166.5 ± 14.9	R	1.94 x 10^6^ ± 5.16 x 10^5^	4.98 x 10^7^ ± 1.68 x 10^7^	2568.2 ± 534.1
3686	2.48 x 10^6^ ± 1.81 x 10^5^	2.78 x 10^6^ ± 3.50 x 10^5^	3.79 x 10^6^ ± 5.69 x 10^5^	111. 9 ± 7.2	153.3 ± 20.6	R	4.02 x 10^6^ ± 1.52 x 10^5^	4.71 x 10^7^ ± 3.92 x 10^6^	1169.6 ± 82.0
4574	1.73 x 10^6^ ± 1.61 x 10^5^	2.02 x 10^6^ ± 5.80 x 10^5^	3.62 x 10^6^ ± 1.18 x 10^6^	115.5 ± 23.6	206.4± 51.2	R	2.78 x 10^6^ ± 3.25 x 10^5^	6.80 x 10^7^ ± 2.06 x 10^7^	2406.7 ± 469.3

^1^ Normal Human Serum

^2^ Human Inactivated Serum

^3^ Colony Forming Units/ml

^4^RP-Resistance phenotype: R – resistant strain, S – sensitive strain, I – intermediate strain.

**Fig 1:**
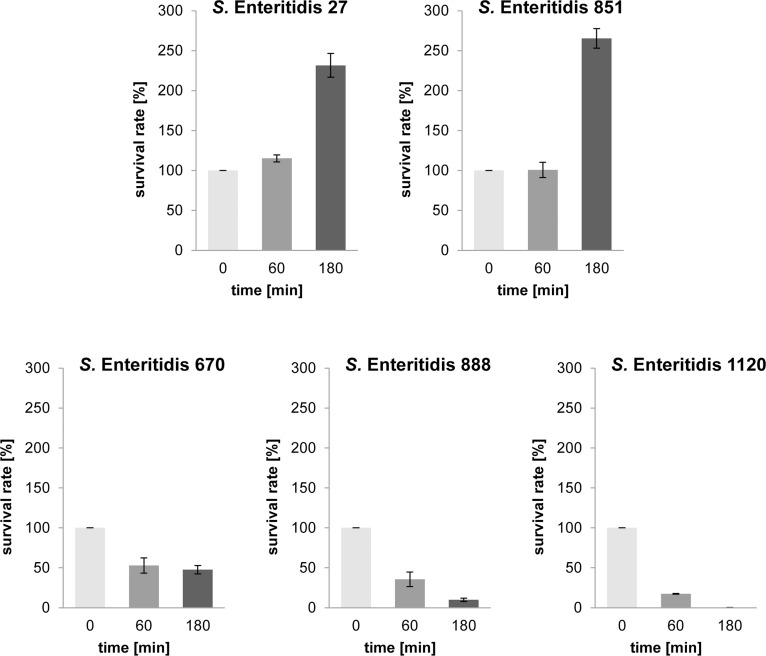
Bactericidal activity of 75% NHS against *S*. Enteritidis strains. Two strains, i.e. *S*. Enteritidis 27, *S*. Enteritidis 851, were resistant to 75% NHS. Three strains, i.e. *S*. Enteritidis 670, *S*. Enteritidis 888 and *S*. Enteritidis 1120, were sensitive to 75% NHS.

Further analyses showed that resistant strains from the same *Salmonella* serovar exhibit extremely different reactions to the bactericidal action of NHS after the challenge with a higher concentration of NHS: after a 180 min incubation in 75% NHS the strains revealed significant differences in the level of their resistance (Table 3). Five extreme strains were used for further experiments. Two strains, i.e. *S*. Enteritidis 27 and *S*. Enteritidis 851, were resistant to 75% NHS (226.6% and 271.9% survival rate after a 180 min cell incubation in serum, respectively), and three strains, i.e. *S*. Enteritidis 670, *S*. Enteritidis 888 and *S*. Enteritidis 1220, were sensitive (44.5%, 8.8% and 0.2% survival rate, respectively). When the bactericidal activity of NHS was heat-inhibited (HIS, control of experiments), bacterial cells proliferated very intensively and all of the tested strains were resistant to 75% HIS (Table 3, Fig 1).

**Table 3:** Bactericidal activity of 75% NHS and 75% HIS decomplemented by heating at 56°C for 30 min against *S*. Enteritidis strains.

Bactericidal activity of 75% NHS^1^								Bactericidal activity of 75% HIS^2^		
Strain	CFU/ml^3^				Survival rateof cells at T_1_ [%]	Survival rateof cells at T_3_ [%]	RP^4^	CFU/ml		Survival rate of cells at T_3_ [%]
	T_0_	T_1_		T_3_				T_0_	T_3_	
27	1.96 x 10^6^ ± 1.59 x 10^5^	2.26 x 10^6^ ± 1.85 x 10^5^	4.54 x 10^6^ ± 3.36 x 10^5^		115.2 ± 4.5	231.7 ± 14.8	R	2.04 x 10^6^ ± 2.52 x 10^5^	1.63 x 10^8^ ± 2.99 x 10^7^	8071.5 ± 1720.1
851	1.47 x 10^6^ ± 1.46 x 10^5^	1.49 x 10^6^ ± 2.25 x 10^5^	3.90 x 10^6^ ± 2.40 x 10^5^		100.7 ± 9.5	265.4 ± 12.2	R	1.54 x 10^6^ ± 2.14 x 10^5^	2.23 x 10^7^ ± 4.89 x 10^6^	1446.8 ± 281.5
670	4.93 x 10^6^ ± 9.56 x 10^5^	2.54 x 10^6^ ± 5.20 x 10^4^	2.31 x 10^6^ ± 2.23 x 10^5^		52.7 ± 9.5	47.5 ± 5.2	S	2.00 x 10^6^ ± 1.51 x 10^5^	3.64 x 10^7^ ± 6.66 x 10^6^	1809.9 ± 227.1
888	5.59 x 10^6^ ± 1.32 x 10^6^	1.91 x 10^6^ ± 1.31 x 10^5^	5.35 x 10^5^ ± 5.46 x 10^4^		35.6 ± 9.1	9.9 ± 2.0	S	1.90 x 10^6^ ± 4.01 x 10^5^	2.14 x 10^7^ ± 5.39 x 10^6^	1122.8 ± 149.0
1120	1.55 x 10^6^ ± 1.10 x 10^5^	2.69 x 10^5^ ± 1.49 x 10^4^	3.36 x 10^3^ ± 8.50 x 10^2^		17.4 ± 0.4	0.2 ± 0.1	S	2.48 x 10^6^ ± 1.92 x 10^5^	1.25 x 10^7^ ± 4.50 x 10^6^	497.3 ± 150.5
351	1.51 x 10^6^ ± 4.66 x 10^5^	1.21 x 10^6^ ± 3.53 x 10^5^	1.18 x 10^6^ ± 1.18 x 10^5^		80.8 ± 2.6	81.6 ± 18,0	I	4.83 x 10^6^ ± 5.80 x 10^5^	3.43 x 10^7^ ± 7.61 x 10^6^	705.8 ± 84.7
920	1.85 x 10^6^ ± 2.59 x 10^5^	1.99 x 10^6^ ± 3.54 x 10^5^	1.30 x 10^6^ ± 2.78 x 10^5^		107.2 ± 7.3	69.9 ± 5.8	I	1.87 x 10^6^ ± 2.47 x 10^5^	7.42 x 10^7^ ±1.35 x 10^7^	3981.8 ± 628.9
1227	3.19 x 10^6^ ± 2.75 x 10^5^	1.65 x 10^6^ ± 6.56 x 10^4^	2.82 x 10^6^ ± 3.29 x 10^5^		52.1 ± 6.2	88.2 ± 5.2	I	5.99 x 10^6^ ± 4.94 x 10^5^	3.34 x 10^7^ ± 2.49 x 10^7^	536.6 ± 389.0
3686	2.00 x 10^6^ ± 5.06 x 10^5^	1.87 x 10^6^ ± 4.31 x 10^5^	1.57 x 10^6^ ± 3.72 x 10^5^		94.2 ± 4.2	78.8 ± 1.2	I	2.01 x 10^6^ ± 4.92 x 10^5^	1.67 x 10^7^ ± 4.61 x 10^6^	850.3 ± 248.8
4574	1.71 x 10^6^ ± 3.93 x 10^5^	1.64 x 10^6^ ± 5.66 x 10^5^	1.53 x 10^6^ ± 2.84 x 10^6^		94.3 ± 10.1	90.4 ± 3.8	I	2.06 x 10^6^ ± 2.48 x 10^5^	4.31 x 10^7^ ± 4.21 x 10^6^	2112.9 ± 341.9

^1^ Normal Human Serum

^2^ Human Inactivated Serum

^3^ Colony Forming Units/ml

^4^RP-Resistance phenotype: R – resistant strain, S – sensitive strain, I – intermediate strain.

Regarding our own results and reports of other research groups, showing that resistance to the bactericidal activity of serum is determined by OMPs and LPS [12, 20, 22, 23], the next stage of research concentrated on the LPS O-antigen chain length distribution and the protein profiles of OMPs.

### SDS-PAGE of LPS

**Fig 2:**
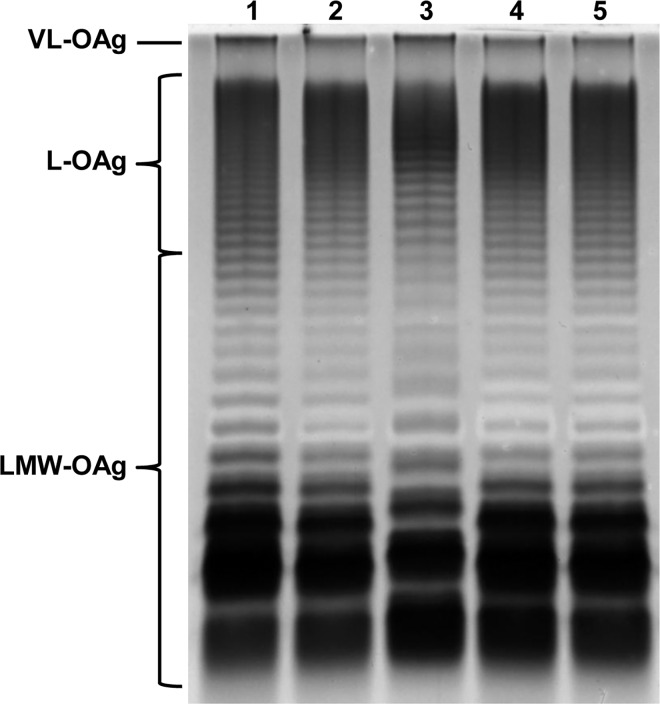
LPS profiles of five extreme strains: Lane 1—*S*. Enteritidis 27, Lane 2—*S*. Enteritidis 851, Lane 3—*S*. Enteritidis 670, Lane 4—*S*. Enteritidis—888, Lane 5—*S*. Enteritidis 1220.

The SDS-PAGE analysis of LPS isolated from bacterial cells showed, that all the five tested strains possessed LPS with a very long O-antigen (VL-OAg LPS, more than 100 O-specific units in the O-chain; Fig 2). That was in accordance with the results of the test with 50% NHS, where all the five strains appeared to be resistant: VL-OAg LPS is described in the literature as an important factor of bacterial resistance to human serum [12]. Although the presence of VL-OAg LPS correlates with the resistance of the strains to 50% NHS, but there is no straight correlation, while 75% NHS has been used for experiments, what indicates the involvement of other factors in the process of bacterial resistance. Additionally the LPS pattern in line 3 (*S*. Enteritidis 670) in the region of LMW-OAg LPS shows distinct differences comparing to the other lines: the difference in the position of respective bands suggests the increase of the molecular mass of the LPS core or O-antigen structure.

### Analysis of 2-DE profiles of isolated OMPs

**Fig 3:**
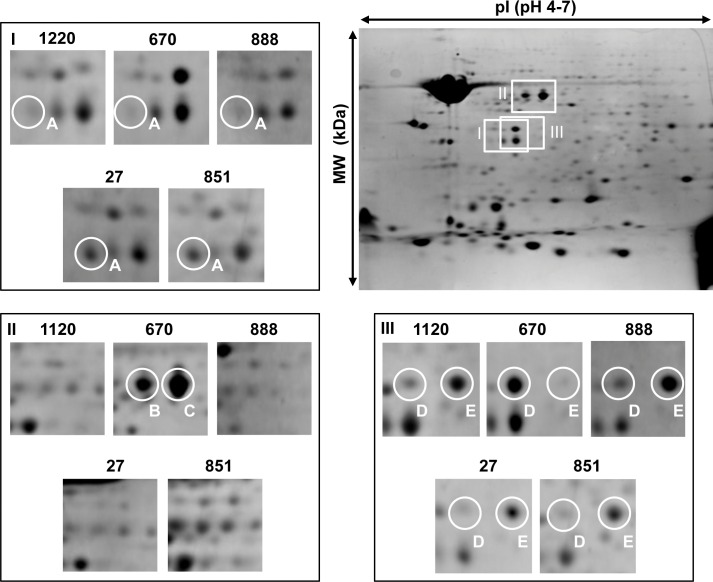
Comparative analysis of OMPs from *S*. Enteritidis strains sensitive and resistant to human serum (75%). Differential expression of marked protein A in serum-sensitive and serum-resistant strains. Both strains resistant to human serum overexpressed protein A (PgtE) (I). The overexpressed proteins labeled as B and C (both identified as FliD) are only present in strain 670 (II). Differential expression of proteins marked as D and E (both identified as OmpA) in serum-sensitive and serum-resistant strains. Strain 670 overexpressed protein D in contrast to the protein labeled with the letter E which is present in lower amounts (III).

We applied a proteomic approach using the 2-DE and mass spectrometry analysis for the identification of specific proteins that could be involved in the phenomenon of serum resistance of these strains. Protein spots on 2-DE were visualized within the molecular weight (MW) range of 10–159 kDa and isoelectric points (pI) of 4–7. In total, 169–180 spots were counted on the maps of *S*. Enteritidis strains by the PDQuest software. The comparative protein pattern analysis of *S*. Enteritidis strains resistant and sensitive to human serum showed differences in the presence of some proteins (Fig 3I–3III). The differential analysis revealed the presence of 5 main protein spots, in which the proteins were subsequently identified (Table 4). MWs of OMPs were distributed in the range of 35.03–49.81 kDa. All the spots were distributed in the range of pI 5.27–5.6. The detailed Mascot search results and MS spectra are provided as supporting information (S1, S2, S3, S4, S5, and S6 Appendices).

**Table 4:** Identification of OMPs from *S*. Enteritidis strains with different sensitivity to 75% serum.

Spots	Identified proteins	Gene symbols	Molecular weight [kDa]	pI	Average sequence coverage [%]
A	Outer membrane protease E	*pgtE*	35.033	5.28	59
B	Flagellar hook-associated protein 2	*fliD*	49.805	5.27	61
C	Flagellar hook-associated protein 2	*fliD*	49.805	5.27	36
D	Outer membrane protein A	*ompA*	37.454	5.6	46
E	Outer membrane protein A	*ompA*	37.454	5.6	30

Outer membrane protease E (PgtE, spot A, Fig 3I) was present in considerably larger quantities in two strains resistant to human serum, i.e. *S*. Enteritidis 27 and *S*. Enteritidis 851.

Flagellar hook associated protein 2 (FliD, spots B and C, Fig 3II) was present in a higher amount in *S*. Enteritidis 670 (strain sensitive to human serum), in contrast to the outer membrane protein A (OmpA, spot E, Fig 3III) which was present in lower amounts in terms of spot intensity in comparison with other strains. In case of OmpA and FliD two post-translational variants were present as two neighboring spots on the gel (spots B and C for FliD, spots D and E for OmpA).

## Discussion

Due to its high virulence, and several features that predispose *S*. Enteritidis to survive and multiply in serum, it is important to explore the role of the surface structures which are involved in the process of avoiding the immune system of the host. All of the tested *S*. Enteritidis strains were resistant to the bactericidal activity of 50% human serum (Table 2), some of the strains presented resistance even to 75% serum (Table 3). Those findings indicate a relatively high incidence of serum-resistant strains in the population of *S*. Enteritidis in Poland.

LPS plays an important role in the pathogenesis of a *Salmonella* infection [8]. Therefore five extreme strains chosen for further experiments after 75% NHS testing were analyzed for their LPS O-specific chains profiles using SDS-PAGE analysis in order to examine the correlation of VL-OAg occurrence with the survival of *Salmonella* in serum. All of the tested strains showed the presence of VL-OAg LPS, which may indicate that the presence of LPS with a very long O-antigen protects a bacterial cell from the action of 50% NHS [14], but for the resistance against 75% NHS the presence of other factors is required. Serum resistance is a multifactorial phenomenon, wherein LPS plays an important role, but the presence of certain OMPs could be decisive.

There are several papers, concerning 2-DE OMP from *Salmonella*, mainly from *S*. Typhimurium strains [25–27]. However, there are no up-to-date data on the 2-DE OMPs analysis from *S*. Enteritidis strains apart from Cho et al. [29]. Therefore a comparative analysis of OMPs from five strains, resistant and sensitive to 75% NHS, was performed. The experiments showed, that the main difference in the OMP composition was a relatively high level of outer membrane protease E (PgtE, spot A, Fig 3I) and a low level of flagellar hook associated protein 2 (FliD, spots B and C, Fig 3II) in resistant strains: *S*. Enteritidis 27 and *S*. Enteritidis 851.

Both proteins take part in the protection of bacterial cells in response to the immunological system activity during the process of pathogenesis. Proteins PgtE from *S*. Typhimurium and Pla from *Yersinia pestis* belong to the enterobacterial outer membrane proteases family called omptins. Omptins are structural homologs possessing a very conservative configuration [22,45,46]. *Salmonella* is a facultative intracellular microorganism preferring the environment of macrophages, where expression of a shortened O-antigen occurs together with PgtE activation [47]. Due to the outer membrane protease E hydrolytic activity of complement components C3, C3b, C4 and C5, this protein is able to mediate in the phenomenon of serum resistance [45, 46]. The finding of our experiments, that two resistant strains have an increased number of PgtE copies in the cell could in consequence have a connection with their special ability to survive in serum.

The *S*. Enteritidis 670 strain differed significantly from the other four analyzed strains. It had the highest level of two variants of flagellar hook associated protein 2 (FliD, spots B and C, Fig 3II) and also of outer membrane protein A (OmpA, spot D, Fig 3III). Protein FliD is a component of bacterial flagella responsible for adhesion. It is essential for the morphogenesis and the elongation of the flagellar filament by facilitating polymerization of the flagellin monomers at the tip of the growing filament. The second protein present in this strain is OmpA—an important structural protein and a receptor for bacteriophages and colicins. Soulas et al. showed that human and mouse macrophages can be activated after contact with OmpA from *K*. *pneumoniae*. They suggested that OmpA recognition by macrophages may initiate an antimicrobial host response (42). Cho et al. analyzing the immunoreactivity of OMPs isolated from *S*. Enteritidis, showed that FliD and OmpA exhibited strong reactivity with serum of chickens immunized with *S*. Enteritidis, which can indicate that the presence of both proteins on the cell surface affects the multiplication in the serum and, consequently, the process of pathogenicity of *Salmonella* [29]. In the analyzed strains, two resistant ones (*S*. Enteritidis 27 and *S*. Enteritidis 851) had a high level of OmpA (spot E, Fig 3III), but a different variant than in *S*. Enteritidis 670 (spot D, Fig 3III). The sensitivity of *S*. Enteritidis 670 could be due to the presence of that OmpA variant in the cell. However, slight differences in the LPS pattern (Fig 2, lane 3) could also be noted, especially in the low molecular weight region.

In conclusion, during present studies on several strains of *S*. Enteritidis we have pinpointed some proteins, that could potentially affect the survival of *Salmonella* strains in serum. In previous results Bugla-Płoskońska et al. [24] presented variability in the survival of *Salmonella* O48 strains after contact with cord serum, where resistant strains exhibited wide variations in OMPs in comparison to susceptible strains. The present findings confirm, that not only the presence of specific proteins but also the presence of their post-translational variants has an influence on the pathogenicity of *S*. Enteritidis.

## Supporting Information

S1 Appendix Mascot search result for protein A (PgtE). (PDF)

S2 Appendix Mascot search result for protein B (FliD). (PDF)

S3 Appendix Mascot search result for protein C (FliD). (PDF)

S4 Appendix Mascot search result for protein D (OmpA). (PDF)

S5 Appendix Mascot search result for protein E (OmpA). (PDF)

S6 Appendix MS spectra. (DOC)
