# Supplementary material for: Proteomic Analysis of Outer Membrane Proteins from Salmonella Enteritidis Strains with Different Sensitivity to Human Serum
Source: PLoS One. 2016 Oct 3;11(10):e0164069. doi: 10.1371/journal.pone.0164069 (PMC5047454; doi:10.1371/journal.pone.0164069)
Supplement: S1 Appendix — (PDF) [file pone.0164069.s001.pdf]

Mascot Search Results

Protein View

Match to: **PGTE_SALTY** Score: **147** Expect: **2.5e-011**
Outer membrane protease E OS=Salmonella typhimurium (strain LT2 / SGSC1412 / ATCC 700720) GN=pgtE PE=3 SV=2

Nominal mass (M_r): **35033**; Calculated pI value: **5.28**
NCBI BLAST search of PGTE_SALTY against nr
Unformatted sequence string for pasting into other applications

Taxonomy: Salmonella enterica subsp. enterica serovar Typhimurium str. LT2

Variable modifications: Carbamidomethyl (C),Oxidation (M)
Cleavage by Trypsin: cuts C-term side of KR unless next residue is P
Number of mass values searched: **69**
Number of mass values matched: **19**
Sequence Coverage: **59%**

Matched peptides shown in **Bold Red**

```
  1 MKKHAIAVMM IAVFSESVYA ESALFIPDVS PDSVTTSLSV GVLNGKSREL
 51 VYDTDTGRKL SQLDWKIKNV ATLQGDLSWE PYSFMTLDAR GWTSLASGSG
101 HMVDHDWMSS EQPGWTDRSI HPDTSVNYAN EYDLNVKGWL LQGDNYKAGV
151 TAGYQETRFS WTARGGSYIY DNGRYIGNFP HGVRGIGYSQ RFEMPYIGLA
201 GDYRINDFEC NVLFKYSDWV NAHDNDEHYM RKLTFREKTE NSRYYGASID
251 AGYYITSNAK IFAEFAYSKY EEGKGGTQII DKTSGDTAYF GGDAAGIANN
301 NYTVTAGLQY RF
```

Show predicted peptides also

Sort Peptides By ◉ Residue Number ○ Increasing Mass ○ Decreasing Mass

Start - End	Observed	Mr(expt)	Mr(calc)	ppm	Miss	Sequence
49 - 58	1168.5525	1167.5452	1167.5408	4	0	R.ELVYDTDTGR.K
49 - 59	1296.6580	1295.6507	1295.6357	12	1	R.ELVYDTDTGRK.L
69 - 90	2514.2189	2513.2116	2513.1897	9	0	K.NVATLQGDLSWEPYSFMTLDAR.G
69 - 90	2530.1897	2529.1824	2529.1846	-1	0	K.NVATLQGDLSWEPYSFMTLDAR.G Oxidation (M)
138 - 147	1193.6054	1192.5981	1192.5877	9	0	K.GWLLQGDNYK.A
148 - 158	1152.5659	1151.5586	1151.5571	1	0	K.AGVTAGYQETR.F
159 - 164	767.3754	766.3681	766.3762	-11	0	R.FSWTAR.G
165 - 174	1101.4869	1100.4796	1100.4887	-8	0	R.GGSYIYDNGR.Y
175 - 184	1159.5862	1158.5790	1158.5934	-12	0	R.YIGNFPHGVR.G
185 - 191	780.3949	779.3876	779.3926	-6	0	R.GIGYSQR.F
192 - 204	1531.7217	1530.7144	1530.7177	-2	0	R.FEMPYIGLAGDYR.I
192 - 204	1547.7058	1546.6985	1546.7126	-9	0	R.FEMPYIGLAGDYR.I Oxidation (M)
205 - 215	1398.6746	1397.6674	1397.6649	2	0	R.INDFECNVLFK.Y Carbamidomethyl (C)
216 - 231	2051.8129	2050.8056	2050.8228	-8	0	K.YSDWVNAHDNDEHYMR.K
216 - 231	2067.8035	2066.7963	2066.8177	-10	0	K.YSDWVNAHDNDEHYMR.K Oxidation (M)
232 - 236	664.4184	663.4111	663.4068	7	1	R.KLTFR.E
244 - 260	1856.8642	1855.8569	1855.8628	-3	0	R.YYGASIDAGYYITSNAK.I
261 - 269	1075.5406	1074.5333	1074.5386	-5	0	K.IFAEFAYSK.Y
283 - 311	2968.3641	2967.3568	2967.3635	-2	0	K.TSGDTAYFGGDAAGIANNNYTVTAGLQYR.F

No match to: 608.3377, 644.0112, 662.2948, 666.0136, 832.2928, 833.0584, 855.0329, 856.5275, 870.5537, 871.0027, 908.5037, [illegible]

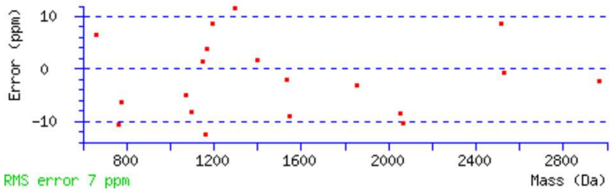


```
ID   PGTE_SALTY              Reviewed;         312 AA.
AC   P06185; Q9KI72;
DT   01-JAN-1988, integrated into UniProtKB/Swiss-Prot.
DT   23-JAN-2002, sequence version 2.
DT   16-MAY-2012, entry version 100.
DE   RecName: Full=Outer membrane protease E;
DE            Short=E protein;
DE            EC=3.4.23.-;
DE   Flags: Precursor;
GN   Name=pgtE; Synonyms=prtA; OrderedLocusNames=STM2395;
OS   Salmonella typhimurium (strain LT2 / SGSC1412 / ATCC 700720).
OC   Bacteria; Proteobacteria; Gammaproteobacteria; Enterobacteriales;
OC   Enterobacteriaceae; Salmonella;
OC   Salmonella enterica subsp. enterica serovar Typhimurium.
OX   NCBI_TaxID=99287;
RN   [1]
RP   NUCLEOTIDE SEQUENCE [GENOMIC DNA].
RX   MEDLINE=87055265; PubMed=3023201; DOI=10.1016/0378-1119(86)90131-9;
RA   Yu G.-Q., Hong J.-S.;
RT   "Identification and nucleotide sequence of the activator gene of the
RT   externally induced phosphoglycerate transport system of Salmonella
RT   typhimurium.";
RL   Gene 45:51-57(1986).
RN   [2]
```

```
RP   NUCLEOTIDE SEQUENCE [GENOMIC DNA].
RX   MEDLINE=20327590; PubMed=10869088;
RX   DOI=10.1128/JB.182.14.4077-4086.2000;
RA   Guina T., Yi E.C., Wang H., Hackett M., Miller S.I.;
RT   "A PhoP-regulated outer membrane protease of Salmonella enterica
RT   serovar typhimurium promotes resistance to alpha-helical antimicrobial
RT   peptides.";
RL   J. Bacteriol. 182:4077-4086(2000).
RN   [3]
RP   NUCLEOTIDE SEQUENCE [LARGE SCALE GENOMIC DNA].
RC   STRAIN=LT2 / SGSC1412 / ATCC 700720;
RX   MEDLINE=21534948; PubMed=11677609; DOI=10.1038/35101614;
RA   McClelland M., Sanderson K.E., Spieth J., Clifton S.W., Latreille P.,
RA   Courtney L., Porwollik S., Ali J., Dante M., Du F., Hou S., Layman D.,
RA   Leonard S., Nguyen C., Scott K., Holmes A., Grewal N., Mulvaney E.,
RA   Ryan E., Sun H., Florea L., Miller W., Stoneking T., Nhan M.,
RA   Waterston R., Wilson R.K.;
RT   "Complete genome sequence of Salmonella enterica serovar Typhimurium
RT   LT2.";
RL   Nature 413:852-856(2001).
RN   [4]
RP   SIMILARITY TO OMPT, AND FUNCTION.
RX   MEDLINE=89213991; PubMed=2651422;
RA   Grodberg J., Dunn J.J.;
RT   "Comparison of Escherichia coli K-12 outer membrane protease OmpT and
RT   Salmonella typhimurium E protein.";
RL   J. Bacteriol. 171:2903-2905(1989).
CC   -!- FUNCTION: Protease that can cleave T7 RNA polymerase. Specific for
CC       paired basic residues.
CC   -!- SUBCELLULAR LOCATION: Cell outer membrane; Multi-pass membrane
CC       protein (By similarity).
CC   -!- SIMILARITY: Belongs to the peptidase A26 family.
CC   -!- SEQUENCE CAUTION:
CC       Sequence=AAA27185.1; Type=Frameshift; Positions=7, 278, 288, 295;
CC   -----------------------------------------------------------------------
CC   Copyrighted by the UniProt Consortium, see http://www.uniprot.org/terms
CC   Distributed under the Creative Commons Attribution-NoDerivs License
CC   -----------------------------------------------------------------------
DR   EMBL; M13923; AAA27185.1; ALT_FRAME; Genomic_DNA.
DR   EMBL; AF239770; AAF85951.1; -; Genomic_DNA.
DR   EMBL; AE006468; AAL21295.1; -; Genomic_DNA.
DR   PIR; B28255; B28255.
DR   RefSeq; NP_461336.1; NC_003197.1.
DR   ProteinModelPortal; P06185; -.
DR   MEROPS; A26.004; -.
DR   PRIDE; P06185; -.
DR   GeneID; 1253917; -.
DR   GenomeReviews; AE006468_GR; STM2395.
DR   KEGG; stm:STM2395; -.
DR   PATRIC; 32383409; VBISalEnt20916_2533.
DR   eggNOG; COG4571; -.
DR   HOGENOM; HOG000117799; -.
DR   KO; K08477; -.
DR   OMA; SQLDWRI; -.
DR   ProtClustDB; PRK10993; -.
DR   BioCyc; STYP99287:STM2395-MONOMER; -.
DR   GO; GO:0009279; C:cell outer membrane; IEA:UniProtKB-SubCell.
DR   GO; GO:0016021; C:integral to membrane; IEA:UniProtKB-KW.
DR   GO; GO:0005886; C:plasma membrane; IEA:UniProtKB-KW.
DR   GO; GO:0004190; F:aspartic-type endopeptidase activity; IEA:UniProtKB-KW.
DR   GO; GO:0006508; P:proteolysis; IEA:UniProtKB-KW.
DR   Gene3D; G3DSA:2.40.128.90; Peptidase_A26; 1.
DR   InterPro; IPR020080; OM_adhesin/peptidase_omptin.
DR   InterPro; IPR023619; Peptidase_A26.
DR   InterPro; IPR020079; Peptidase_A26_CS.
DR   InterPro; IPR000036; Peptidase_A26_omptin.
DR   Pfam; PF01278; Omptin; 1.
DR   PIRSF; PIRSF001522; Peptidase_A26; 1.
DR   PRINTS; PR00482; OMPTIN.
DR   SUPFAM; SSF69917; OM_adhesin/protease_omptin; 1.
DR   PROSITE; PS00834; OMPTIN_1; 1.
DR   PROSITE; PS00835; OMPTIN_2; 1.
PE   3: Inferred from homology;
KW   Aspartyl protease; Cell membrane; Cell outer membrane;
KW   Complete proteome; Hydrolase; Membrane; Protease; Reference proteome;
KW   Signal; Transmembrane; Transmembrane beta strand.
FT   SIGNAL        1     37       Potential.
FT   CHAIN        38    312       Outer membrane protease E.
FT                                /FTId=PRO_0000025818.
FT   ACT_SITE    104    104       By similarity.
FT   ACT_SITE    106    106       By similarity.
FT   ACT_SITE    226    226       By similarity.
FT   ACT_SITE    228    228       By similarity.
FT   CONFLICT    296    296       G -> S (in Ref. 1).
SQ   SEQUENCE   312 AA;  35055 MW;  5A6841732AF2E8C4 CRC64;
     MKKHAIAVMM IAVFSESVYA ESALFIPDVS PDSVTTSLSV GVLNGKSREL VYDTDTGRKL
     SQLDWKIKNV ATLQGDLSWE PYSFMTLDAR GWTSLASGSG HMVDHDWMSS EQPGWTDRSI
     HPDTSVNYAN EYDLNVKGWL LQGDNYKAGV TAGYQETRFS WTARGGSYIY DNGRYIGNFP
     HGVRGIGYSQ RFEMPYIGLA GDYRINDFEC NVLFKYSDWV NAHDNDEHYM RKLTFREKTE
     NSRYYGASID AGYYITSNAK IFAEFAYSKY EEGKGGTQII DKTSGDTAYF GGDAAGIANN
     NYTVTAGLQY RF
```

Mascot: http://www.matrixscience.com/
