# Supplementary material for: Proteomic Analysis of Outer Membrane Proteins from Salmonella Enteritidis Strains with Different Sensitivity to Human Serum
Source: PLoS One. 2016 Oct 3;11(10):e0164069. doi: 10.1371/journal.pone.0164069 (PMC5047454; doi:10.1371/journal.pone.0164069)
Supplement: S2 Appendix — (PDF) [file pone.0164069.s002.pdf]

Mascot Search Results

Protein View

Match to: **FLID_SALTY** Score: **135** Expect: **4e-010**
Flagellar hook-associated protein 2 OS=Salmonella typhimurium (strain LT2 / SGSC1412 / ATCC 700720) GN=fliD PE=1 SV=2

Nominal mass (M_r): **49805**; Calculated pI value: **5.27**
NCBI BLAST search of FLID_SALTY against nr
Unformatted sequence string for pasting into other applications

Taxonomy: Salmonella enterica subsp. enterica serovar Typhimurium str. LT2

Variable modifications: Carbamidomethyl (C),Oxidation (M)
Cleavage by Trypsin: cuts C-term side of KR unless next residue is P
Number of mass values searched: **87**
Number of mass values matched: **24**
Sequence Coverage: **61%**

Matched peptides shown in **Bold Red**

1 M**ASISSLGVG SNLPLDQLLT DLTK**NEKGRL TPITKQQSAN SAKLTAYGTL
51 KSALEK**FQTA NTALNK**ADLF KSTVASSTTE DLKVSTTAGA AAGTYK**INVT**
101 **QLAAAQSLAT K**TTFATTK**EQ LGDTSVTSRT** IK**IEQPGR**KE PLEIKLDK**GD**
151 **TSMEAIRDAI NDADSGIAAS IVK**VKENEFQ LVLTANSGTD NTMKITVEGD
201 TK**LNDLLAYD STTNTGNMQE LVK**AENAK**LN VNGIDIERQS NTVTDAPQGI**
251 **TLTLTK**K**VTD ATVTVTKDDT** KAKEAIK**SWV DAYNSLVDTF SSLTK**YTAVE
301 PGEEASDK**NG ALLGDSVVRT IQTGIRAQFA NSGSNSAFKT MAEIGITQDG**
351 **TSGKLK**IDDD KLTKVLKDNT AAARELLVGD GKETGITTKI ATEVK**SYLAD**
401 **DGIIDNAQDN VNATLK**SLTK **QYLSVSNSID ETVARYKAQF TQLDTMMSKL**
451 **NNTSSYLTQQ FTAMNK**S

Show predicted peptides also

Sort Peptides By ◉ Residue Number ○ Increasing Mass ○ Decreasing Mass

Start - End	Observed	Mr(expt)	Mr(calc)	ppm	Miss	Sequence
2 - 24	2342.2781	2341.2708	2341.2740	-1	0	M.ASISSLGVGSNLPLDQLLTDLTK.N
57 - 66	1107.5633	1106.5560	1106.5720	-14	0	K.FQTANTALNK.A
97 - 111	1528.8687	1527.8614	1527.8620	-0	0	K.INVTQLAAAQSLATK.T
119 - 129	1192.5864	1191.5791	1191.5732	5	0	K.EQLGDTSVTSR.T
133 - 138	699.3677	698.3604	698.3711	-15	0	K.IEQPGR.K
149 - 173	2520.2265	2519.2192	2519.2173	1	1	K.GDTSMEAIRDAINDADSGIAASIVK.V
149 - 173	2536.2191	2535.2118	2535.2122	-0	1	K.GDTSMEAIRDAINDADSGIAASIVK.V Oxidation (M)
203 - 223	2340.1412	2339.1340	2339.1315	1	0	K.LNDLLAYDSTTNTGNMQELVK.A
203 - 223	2356.1374	2355.1302	2355.1264	2	0	K.LNDLLAYDSTTNTGNMQELVK.A Oxidation (M)
229 - 238	1142.6162	1141.6089	1141.6091	-0	0	K.LNVNGIDIER.Q
239 - 256	1887.9987	1886.9915	1886.9949	-2	0	R.QSNTVTDAPQGITLTLTK.K
258 - 271	1493.7317	1492.7245	1492.7621	-25	1	K.VTDATVTVTKDDTK.A
278 - 295	2032.9838	2031.9765	2031.9789	-1	0	K.SWVDAYNSLVDTFSSLTK.Y
309 - 319	1100.6040	1099.5968	1099.5986	-2	0	K.NGALLGDSVVR.T
320 - 326	788.4555	787.4482	787.4552	-9	0	R.TIQTGIR.A
327 - 339	1328.6252	1327.6179	1327.6157	2	0	R.AQFANSGSNSAFK.T
340 - 356	1765.7321	1764.7249	1764.8928	-95	1	K.TMAEIGITQDGTSGKLK.I Oxidation (M)
396 - 416	2250.0839	2249.0766	2249.0811	-2	0	K.SYLADDGIIDNAQDNVNATLK.S
421 - 435	1681.8376	1680.8304	1680.8319	-1	0	K.QYLSVSNSIDETVAR.Y
436 - 449	1707.7717	1706.7644	1706.8008	-21	1	R.YKAQFTQLDTMMSK.L Oxidation (M)
438 - 449	1400.6553	1399.6480	1399.6476	0	0	K.AQFTQLDTMMSK.L
438 - 449	1416.6540	1415.6468	1415.6425	3	0	K.AQFTQLDTMMSK.L Oxidation (M)
450 - 466	1960.9385	1959.9312	1959.9360	-2	0	K.LNNTSSYLTQQFTAMNK.S
450 - 466	1976.9327	1975.9255	1975.9309	-3	0	K.LNNTSSYLTQQFTAMNK.S Oxidation (M)

No match to: 634.2548, 644.0073, 662.2896, 832.3028, 855.0469, 881.2513, 1036.5412, 1060.0607, 1109.5032, 1143.6116, 1193.6

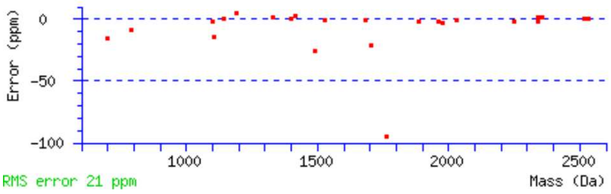


```
ID   FLID_SALTY              Reviewed;         467 AA.
AC   P16328;
DT   01-AUG-1990, integrated into UniProtKB/Swiss-Prot.
DT   23-JAN-2007, sequence version 2.
DT   16-MAY-2012, entry version 81.
DE   RecName: Full=Flagellar hook-associated protein 2;
DE            Short=HAP2;
DE   AltName: Full=Filament cap protein;
DE   AltName: Full=Flagellar cap protein;
GN   Name=fliD; Synonyms=flaV, flbC; OrderedLocusNames=STM1960;
OS   Salmonella typhimurium (strain LT2 / SGSC1412 / ATCC 700720).
OC   Bacteria; Proteobacteria; Gammaproteobacteria; Enterobacteriales;
OC   Enterobacteriaceae; Salmonella;
OC   Salmonella enterica subsp. enterica serovar Typhimurium.
OX   NCBI_TaxID=99287;
RN   [1]
```

```
RP   NUCLEOTIDE SEQUENCE [GENOMIC DNA].
RX   MEDLINE=90294298; PubMed=2193164; DOI=10.1016/S0022-2836(05)80266-9;
RA   Homma M., Derosier D.J., Macnab R.M.;
RT   "Flagellar hook and hook-associated proteins of Salmonella typhimurium
RT   and their relationship to other axial components of the flagellum.";
RL   J. Mol. Biol. 213:819-832(1990).
RN   [2]
RP   NUCLEOTIDE SEQUENCE [LARGE SCALE GENOMIC DNA].
RC   STRAIN=LT2 / SGSC1412 / ATCC 700720;
RX   MEDLINE=21534948; PubMed=11677609; DOI=10.1038/35101614;
RA   McClelland M., Sanderson K.E., Spieth J., Clifton S.W., Latreille P.,
RA   Courtney L., Porwollik S., Ali J., Dante M., Du F., Hou S., Layman D.,
RA   Leonard S., Nguyen C., Scott K., Holmes A., Grewal N., Mulvaney E.,
RA   Ryan E., Sun H., Florea L., Miller W., Stoneking T., Nhan M.,
RA   Waterston R., Wilson R.K.;
RT   "Complete genome sequence of Salmonella enterica serovar Typhimurium
RT   LT2.";
RL   Nature 413:852-856(2001).
RN   [3]
RP   NUCLEOTIDE SEQUENCE [GENOMIC DNA] OF 441-467.
RC   STRAIN=SJW1103;
RX   MEDLINE=92407478; PubMed=1527488;
RA   Kawagishi I., Mueller V., Williams A.W., Irikura V.M., Macnab R.M.;
RT   "Subdivision of flagellar region III of the Escherichia coli and
RT   Salmonella typhimurium chromosomes and identification of two
RT   additional flagellar genes.";
RL   J. Gen. Microbiol. 138:1051-1065(1992).
RN   [4]
RP   SUBUNIT.
RX   MEDLINE=99096876; PubMed=9878359; DOI=10.1006/jmbi.1998.2274;
RA   Vonderviszt F., Imada K., Furukawa Y., Uedaira H., Taniguchi H.,
RA   Namba K.;
RT   "Mechanism of self-association and filament capping by flagellar
RT   HAP2.";
RL   J. Mol. Biol. 284:1399-1416(1998).
RN   [5]
RP   SUBUNIT.
RX   MEDLINE=20568409; PubMed=11118149; DOI=10.1126/science.290.5499.2148;
RA   Yonekura K., Maki S., Morgan D.G., DeRosier D.J., Vonderviszt F.,
RA   Imada K., Namba K.;
RT   "The bacterial flagellar cap as the rotary promoter of flagellin self-
RT   assembly.";
RL   Science 290:2148-2152(2000).
CC   -!- FUNCTION: Required for the morphogenesis and for the elongation of
CC       the flagellar filament by facilitating polymerization of the
CC       flagellin monomers at the tip of growing filament. Forms a capping
CC       structure, which prevents flagellin subunits (transported through
CC       the central channel of the flagellum) from leaking out without
CC       polymerization at the distal end.
CC   -!- SUBUNIT: Homopentamer.
CC   -!- SUBCELLULAR LOCATION: Secreted. Bacterial flagellum.
CC   -!- SIMILARITY: Belongs to the fliD family.
CC   -----------------------------------------------------------------------
CC   Copyrighted by the UniProt Consortium, see http://www.uniprot.org/terms
CC   Distributed under the Creative Commons Attribution-NoDerivs License
CC   -----------------------------------------------------------------------
DR   EMBL; X51740; CAA36030.1; -; Genomic_DNA.
DR   EMBL; AE006468; AAL20872.1; -; Genomic_DNA.
DR   EMBL; M85241; AAA27076.1; -; Genomic_DNA.
DR   PIR; S10364; S10364.
DR   RefSeq; NP_460913.1; NC_003197.1.
DR   ProteinModelPortal; P16328; -.
DR   PRIDE; P16328; -.
DR   GeneID; 1253481; -.
DR   GenomeReviews; AE006468_GR; STM1960.
DR   KEGG; stm:STM1960; -.
DR   PATRIC; 32382481; VBISalEnt20916_2076.
DR   eggNOG; COG1345; -.
DR   HOGENOM; HOG000254632; -.
DR   KO; K02407; -.
DR   OMA; FRTQISA; -.
DR   ProtClustDB; PRK08032; -.
DR   BioCyc; STYP99287:STM1960-MONOMER; -.
DR   GO; GO:0009288; C:bacterial-type flagellum; IEA:UniProtKB-SubCell.
DR   GO; GO:0005576; C:extracellular region; IEA:UniProtKB-SubCell.
DR   GO; GO:0007155; P:cell adhesion; IEA:InterPro.
DR   GO; GO:0006928; P:cellular component movement; IEA:InterPro.
DR   GO; GO:0009296; P:flagellum assembly; IEA:InterPro.
DR   InterPro; IPR010810; Flagellin_hook_IN_motif.
DR   InterPro; IPR010809; FliD_C.
DR   InterPro; IPR003481; FliD_N.
DR   Pfam; PF07196; Flagellin_IN; 1.
DR   Pfam; PF07195; FliD_C; 1.
DR   Pfam; PF02465; FliD_N; 1.
PE   1: Evidence at protein level;
KW   Bacterial flagellum; Coiled coil; Complete proteome;
KW   Reference proteome; Secreted.
FT   INIT_MET      1      1       Removed.
FT   CHAIN         2    467       Flagellar hook-associated protein 2.
FT                                /FTId=PRO_0000177024.
FT   COILED      411    439       Potential.
SQ   SEQUENCE   467 AA;  49835 MW;  5C1BD69A1F233BEA CRC64;
     MASISSLGVG SNLPLDQLLT DLTKNEKGRL TPITKQQSAN SAKLTAYGTL KSALEKFQTA
     NTALNKADLF KSTVASSTTE DLKVSTTAGA AAGTYKINVT QLAAAQSLAT KTTFATTKEQ
     LGDTSVTSRT IKIEQPGRKE PLEIKLDKGD TSMEAIRDAI NDADSGIAAS IVKVKENEFQ
     LVLTANSGTD NTMKITVEGD TKLNDLLAYD STTNTGNMQE LVKAENAKLN VNGIDIERQS
     NTVTDAPQGI TLTLTKKVTD ATVTVTKDDT KAKEAIKSWV DAYNSLVDTF SSLTKYTAVE
     PGEEASDKNG ALLGDSVVRT IQTGIRAQFA NSGSNSAFKT MAEIGITQDG TSGKLKIDDD
     KLTKVLKDNT AAARELLVGD GKETGITTKI ATEVKSYLAD DGIIDNAQDN VNATLKSLTK
```

```
QYLSVSNSID ETVARYKAQF TQLDTMMSKL NNTSSYLTQQ FTAMNKS
```

**Mascot:** http://www.matrixscience.com/
