# Supplementary material for: Proteomic Analysis of Outer Membrane Proteins from Salmonella Enteritidis Strains with Different Sensitivity to Human Serum
Source: PLoS One. 2016 Oct 3;11(10):e0164069. doi: 10.1371/journal.pone.0164069 (PMC5047454; doi:10.1371/journal.pone.0164069)
Supplement: S3 Appendix — (PDF) [file pone.0164069.s003.pdf]

Mascot Search Results

Protein View

Match to: **FLID_SALTY** Score: **98** Expect: **1.9e-006**
Flagellar hook-associated protein 2 OS=Salmonella typhimurium (strain LT2 / SGSC1412 / ATCC 700720) GN=fliD PE=1 SV=2

Nominal mass (M_r): **49805**; Calculated pI value: **5.27**
NCBI BLAST search of FLID_SALTY against nr
Unformatted sequence string for pasting into other applications

Taxonomy: Salmonella enterica subsp. enterica serovar Typhimurium str. LT2

Variable modifications: Carbamidomethyl (C),Oxidation (M)
Cleavage by Trypsin: cuts C-term side of KR unless next residue is P
Number of mass values searched: **50**
Number of mass values matched: **13**
Sequence Coverage: **36%**

Matched peptides shown in **Bold Red**

1 MASISSLGVG SNLPLDQLLT DLTKNEKGRL TPITKQQSAN SAKLTAYGTL
51 KSALEK**FQTA** **NTALNK**ADLF KSTVASSTTE DLKVSTTAGA AAGTYK**INVT**
101 **QLAAAQSLAT** **K**TTFATTKEQ LGDTSVTSRT IKIEQPGRKE PLEIKLDKGD
151 TSMEAIRDAI NDADSGIAAS IVK**VKENEFQ** **LVLTANSGTD** **NTMK**ITVEGD
201 TKLNDLLAYD STTNTGNMQE LVKAENAK**LN** **VNGIDIER**QS NTVTDAPQGI
251 TLTLTKK**VTD** **ATVTVTKDDT** KAKEAIK**SWV** **DAYNSLVDTF** **SSLTK**YTAVE
301 PGEEASDK**NG** **ALLGDSVVRT** **IQTGIRAQFA** **NSGSNSAFKT** MAEIGITQDG
351 TSGKLKIDDD KLTKVLKDNT AAARELLVGD GKETGITTKI ATEVK**SYLAD**
401 **DGIIDNAQDN** **VNATLK**SLTK **QYLSVSNSID** **ETVARYKAQF** **TQLDTMMSK**L
451 NNTSSYLTQQ FTAMNKS

Show predicted peptides also

Sort Peptides By ◉ Residue Number ○ Increasing Mass ○ Decreasing Mass

Start - End	Observed	Mr(expt)	Mr(calc)	ppm	Miss	Sequence	
57 - 66	1107.5593	1106.5520	1106.5720	-18	0	K.FQTANTALNK.A	
97 - 111	1528.8756	1527.8684	1527.8620	4	0	K.INVTQLAAAQSLATK.T	
174 - 194	2339.1859	2338.1786	2338.1475	13	1	K.VKENEFQLVLTANSGTDNTMK.I	
174 - 194	2355.1866	2354.1793	2354.1424	16	1	K.VKENEFQLVLTANSGTDNTMK.I	Oxidation (M)
229 - 238	1142.6248	1141.6176	1141.6091	7	0	K.LNVNGIDIER.Q	
258 - 271	1493.7333	1492.7260	1492.7621	-24	1	K.VTDATVTVTKDDTK.A	
278 - 295	2032.9860	2031.9787	2031.9789	-0	0	K.SWVDAYNSLVDTFSSLTK.Y	
309 - 319	1100.6029	1099.5956	1099.5986	-3	0	K.NGALLGDSVVR.T	
320 - 326	788.4559	787.4487	787.4552	-8	0	R.TIQTGIR.A	
327 - 339	1328.6267	1327.6194	1327.6157	3	0	R.AQFANSGSNSAFK.T	
396 - 416	2250.1251	2249.1178	2249.0811	16	0	K.SYLADDGIIDNAQDNVNATLK.S	
421 - 435	1681.8403	1680.8330	1680.8319	1	0	K.QYLSVSNSIDETVAR.Y	
436 - 449	1707.7696	1706.7623	1706.8008	-23	1	R.YKAQFTQLDTMMSK.L	Oxidation (M)

No match to: 628.0453, 644.0066, 650.0311, 662.2878, 666.0072, 832.2875, 833.0613, 855.0439, 870.5380, 871.0200, 973.5341, [illegible]

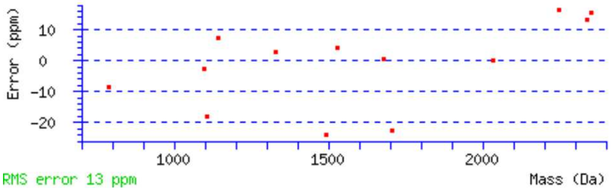


```
ID   FLID_SALTY              Reviewed;         467 AA.
AC   P16328;
DT   01-AUG-1990, integrated into UniProtKB/Swiss-Prot.
DT   23-JAN-2007, sequence version 2.
DT   16-MAY-2012, entry version 81.
DE   RecName: Full=Flagellar hook-associated protein 2;
DE            Short=HAP2;
DE   AltName: Full=Filament cap protein;
DE   AltName: Full=Flagellar cap protein;
GN   Name=fliD; Synonyms=flaV, flbC; OrderedLocusNames=STM1960;
OS   Salmonella typhimurium (strain LT2 / SGSC1412 / ATCC 700720).
OC   Bacteria; Proteobacteria; Gammaproteobacteria; Enterobacteriales;
OC   Enterobacteriaceae; Salmonella;
OC   Salmonella enterica subsp. enterica serovar Typhimurium.
OX   NCBI_TaxID=99287;
RN   [1]
RP   NUCLEOTIDE SEQUENCE [GENOMIC DNA].
RX   MEDLINE=90294298; PubMed=2193164; DOI=10.1016/S0022-2836(05)80266-9;
RA   Homma M., Derosier D.J., Macnab R.M.;
RT   "Flagellar hook and hook-associated proteins of Salmonella typhimurium
RT   and their relationship to other axial components of the flagellum.";
RL   J. Mol. Biol. 213:819-832(1990).
RN   [2]
RP   NUCLEOTIDE SEQUENCE [LARGE SCALE GENOMIC DNA].
RC   STRAIN=LT2 / SGSC1412 / ATCC 700720;
RX   MEDLINE=21534948; PubMed=11677609; DOI=10.1038/35101614;
RA   McClelland M., Sanderson K.E., Spieth J., Clifton S.W., Latreille P.,
RA   Courtney L., Porwollik S., Ali J., Dante M., Du F., Hou S., Layman D.,
```

```
RA   Leonard S., Nguyen C., Scott K., Holmes A., Grewal N., Mulvaney E.,
RA   Ryan E., Sun H., Florea L., Miller W., Stoneking T., Nhan M.,
RA   Waterston R., Wilson R.K.;
RT   "Complete genome sequence of Salmonella enterica serovar Typhimurium
RT   LT2.";
RL   Nature 413:852-856(2001).
RN   [3]
RP   NUCLEOTIDE SEQUENCE [GENOMIC DNA] OF 441-467.
RC   STRAIN=SJW1103;
RX   MEDLINE=92407478; PubMed=1527488;
RA   Kawagishi I., Mueller V., Williams A.W., Irikura V.M., Macnab R.M.;
RT   "Subdivision of flagellar region III of the Escherichia coli and
RT   Salmonella typhimurium chromosomes and identification of two
RT   additional flagellar genes.";
RL   J. Gen. Microbiol. 138:1051-1065(1992).
RN   [4]
RP   SUBUNIT.
RX   MEDLINE=99096876; PubMed=9878359; DOI=10.1006/jmbi.1998.2274;
RA   Vonderviszt F., Imada K., Furukawa Y., Uedaira H., Taniguchi H.,
RA   Namba K.;
RT   "Mechanism of self-association and filament capping by flagellar
RT   HAP2.";
RL   J. Mol. Biol. 284:1399-1416(1998).
RN   [5]
RP   SUBUNIT.
RX   MEDLINE=20568409; PubMed=11118149; DOI=10.1126/science.290.5499.2148;
RA   Yonekura K., Maki S., Morgan D.G., DeRosier D.J., Vonderviszt F.,
RA   Imada K., Namba K.;
RT   "The bacterial flagellar cap as the rotary promoter of flagellin self-
RT   assembly.";
RL   Science 290:2148-2152(2000).
CC   -!- FUNCTION: Required for the morphogenesis and for the elongation of
CC       the flagellar filament by facilitating polymerization of the
CC       flagellin monomers at the tip of growing filament. Forms a capping
CC       structure, which prevents flagellin subunits (transported through
CC       the central channel of the flagellum) from leaking out without
CC       polymerization at the distal end.
CC   -!- SUBUNIT: Homopentamer.
CC   -!- SUBCELLULAR LOCATION: Secreted. Bacterial flagellum.
CC   -!- SIMILARITY: Belongs to the fliD family.
CC   -----------------------------------------------------------------------
CC   Copyrighted by the UniProt Consortium, see http://www.uniprot.org/terms
CC   Distributed under the Creative Commons Attribution-NoDerivs License
CC   -----------------------------------------------------------------------
DR   EMBL; X51740; CAA36030.1; -; Genomic_DNA.
DR   EMBL; AE006468; AAL20872.1; -; Genomic_DNA.
DR   EMBL; M85241; AAA27076.1; -; Genomic_DNA.
DR   PIR; S10364; S10364.
DR   RefSeq; NP_460913.1; NC_003197.1.
DR   ProteinModelPortal; P16328; -.
DR   PRIDE; P16328; -.
DR   GeneID; 1253481; -.
DR   GenomeReviews; AE006468_GR; STM1960.
DR   KEGG; stm:STM1960; -.
DR   PATRIC; 32382481; VBISalEnt20916_2076.
DR   eggNOG; COG1345; -.
DR   HOGENOM; HOG000254632; -.
DR   KO; K02407; -.
DR   OMA; FRTQISA; -.
DR   ProtClustDB; PRK08032; -.
DR   BioCyc; STYP99287:STM1960-MONOMER; -.
DR   GO; GO:0009288; C:bacterial-type flagellum; IEA:UniProtKB-SubCell.
DR   GO; GO:0005576; C:extracellular region; IEA:UniProtKB-SubCell.
DR   GO; GO:0007155; P:cell adhesion; IEA:InterPro.
DR   GO; GO:0006928; P:cellular component movement; IEA:InterPro.
DR   GO; GO:0009296; P:flagellum assembly; IEA:InterPro.
DR   InterPro; IPR010810; Flagellin_hook_IN_motif.
DR   InterPro; IPR010809; FliD_C.
DR   InterPro; IPR003481; FliD_N.
DR   Pfam; PF07196; Flagellin_IN; 1.
DR   Pfam; PF07195; FliD_C; 1.
DR   Pfam; PF02465; FliD_N; 1.
PE   1: Evidence at protein level;
KW   Bacterial flagellum; Coiled coil; Complete proteome;
KW   Reference proteome; Secreted.
FT   INIT_MET      1      1       Removed.
FT   CHAIN         2    467       Flagellar hook-associated protein 2.
FT                                /FTId=PRO_0000177024.
FT   COILED      411    439       Potential.
SQ   SEQUENCE   467 AA;  49835 MW;  5C1BD69A1F233BEA CRC64;
     MASISSLGVG SNLPLDQLLT DLTKNEKGRL TPITKQQSAN SAKLTAYGTL KSALEKFQTA
     NTALNKADLF KSTVASSTTE DLKVSTTAGA AAGTYKINVT QLAAAQSLAT KTTFATTKEQ
     LGDTSVTSRT IKIEQPGRKE PLEIKLDKGD TSMEAIRDAI NDADSGIAAS IVKVKENEFQ
     LVLTANSGTD NTMKITVEGD TKLNDLLAYD STTNTGNMQE LVKAENAKLN VNGIDIERQS
     NTVTDAPQGI TLTLTKKVTD ATVTVTKDDT KAKEAIKSWV DAYNSLVDTF SSLTKYTAVE
     PGEEASDKNG ALLGDSVVRT IQTGIRAQFA NSGSNSAFKT MAEIGITQDG TSGKLKIDDD
     KLTKVLKDNT AAARELLVGD GKETGITTKI ATEVKSYLAD DGIIDNAQDN VNATLKSLTK
     QYLSVSNSID ETVARYKAQF TQLDTMMSKL NNTSSYLTQQ FTAMNKS
```

Mascot: http://www.matrixscience.com/
