# Supplementary material for: Proteomic Analysis of Outer Membrane Proteins from Salmonella Enteritidis Strains with Different Sensitivity to Human Serum
Source: PLoS One. 2016 Oct 3;11(10):e0164069. doi: 10.1371/journal.pone.0164069 (PMC5047454; doi:10.1371/journal.pone.0164069)
Supplement: S4 Appendix — (PDF) [file pone.0164069.s004.pdf]

Mascot Search Results

Protein View

Match to: **OMPA_SALTY** Score: **138** Expect: **2e-010**
Outer membrane protein A OS=Salmonella typhimurium (strain LT2 / SGSC1412 / ATCC 700720) GN=ompA PE=3 SV=2

Nominal mass (M_r): **37492**; Calculated pI value: **5.60**
NCBI BLAST search of OMPA_SALTY against nr
Unformatted sequence string for pasting into other applications

Taxonomy: Salmonella enterica subsp. enterica serovar Typhimurium str. LT2

Variable modifications: Carbamidomethyl (C),Oxidation (M)
Cleavage by Trypsin: cuts C-term side of KR unless next residue is P
Number of mass values searched: **45**
Number of mass values matched: **16**
Sequence Coverage: **46%**

Matched peptides shown in **Bold Red**

1 MKKTAIAIAV ALAGFATVAQ AAPK**DNTWYA** **GAK**LGWSQYH DTGFIHNDGP
51 THENQLGAGA FGGYQVNPYV GFEMGYDWLG RMPYKGDNIN GAYKAQGVQL
101 TAK**LGYPITD** **DLDVYTRLGG** **MVWR**ADTKSN VPGGPSTK**DH** **DTGVSPVFAG**
151 **GIEYAITPEI** **ATR**LEYQWTN NIGDANTIGT RPDNGLLSVG VSYR**FGQQEA**
201 **APVVAPAPAP** **APEVQTK**HFT LK**SDVLFNFN** **K**STLKPEGQQ ALDQLYSQLS
251 NLDPK**DGSVV** **VLGFTDRIGS** **DAYNQGLSEK** **RAQSVVDYLI** **SK**GIPSDKIS
301 AR**GMGESNPV** **TGNTCDNVKP** **RAALIDCLAP** **DRRVEIEVKG** VKDVVTQPQA
351

Show predicted peptides also

Sort Peptides By ◉ Residue Number ○ Increasing Mass ○ Decreasing Mass

Start - End	Observed	Mr(expt)	Mr(calc)	ppm	Miss	Sequence
25 - 33	1025.4610	1024.4537	1024.4614	-7	0	K.DNTWYAGAK.L
104 - 117	1640.7993	1639.7920	1639.8094	-11	0	K.LGYPITDDLDVYTR.L
118 - 124	818.4228	817.4155	817.4269	-14	0	R.LGGMVWR.A
118 - 124	834.4288	833.4215	833.4218	-0	0	R.LGGMVWR.A Oxidation (M)
139 - 163	2616.5466	2615.5394	2615.2868	97	0	K.DHDTGVSPVFAGGIEYAITPEIATR.L
195 - 217	2303.2975	2302.2902	2302.1957	41	0	R.FGQQEAAPVVAPAPAPAPEVQTK.H
223 - 231	1083.5485	1082.5413	1082.5397	1	0	K.SDVLFNFNK.S
256 - 267	1264.6321	1263.6248	1263.6460	-17	0	K.DGSVVVLGFTDR.I
268 - 280	1381.6573	1380.6500	1380.6521	-1	0	R.IGSDAYNQGLSEK.R
268 - 281	1537.7366	1536.7293	1536.7532	-16	1	R.IGSDAYNQGLSEKR.A
281 - 292	1378.7443	1377.7371	1377.7616	-18	1	K.RAQSVVDYLISK.G
303 - 321	2032.9644	2031.9572	2031.9102	23	0	R.GMGESNPVTGNTCDNVKPR.A Carbamidomethyl (C)
322 - 332	1157.5886	1156.5813	1156.5910	-8	0	R.AALIDCLAPDR.R
322 - 332	1214.6054	1213.5982	1213.6125	-12	0	R.AALIDCLAPDR.R Carbamidomethyl (C)
322 - 333	1370.6942	1369.6869	1369.7136	-19	1	R.AALIDCLAPDRR.V Carbamidomethyl (C)
333 - 339	872.4874	871.4801	871.5127	-37	1	R.RVEIEVK.G

No match to: 608.3076, 643.9765, 662.2539, 832.2619, 833.0444, 855.0124, 870.9773, 973.5256, 1060.0080, 1065.5099, 1193.597[illegible]

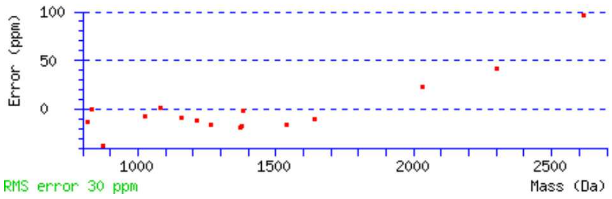

```
ID   OMPA_SALTY              Reviewed;         350 AA.
AC   P02936;
DT   21-JUL-1986, integrated into UniProtKB/Swiss-Prot.
DT   23-JAN-2002, sequence version 2.
DT   16-MAY-2012, entry version 100.
DE   RecName: Full=Outer membrane protein A;
DE   AltName: Full=Outer membrane major heat-modifiable protein;
DE   AltName: Full=Outer membrane protein 33K;
DE   Flags: Precursor;
GN   Name=ompA; OrderedLocusNames=STM1070;
OS   Salmonella typhimurium (strain LT2 / SGSC1412 / ATCC 700720).
OC   Bacteria; Proteobacteria; Gammaproteobacteria; Enterobacteriales;
OC   Enterobacteriaceae; Salmonella;
OC   Salmonella enterica subsp. enterica serovar Typhimurium.
OX   NCBI_TaxID=99287;
RN   [1]
RP   NUCLEOTIDE SEQUENCE [GENOMIC DNA].
RX   MEDLINE=83287368; PubMed=6349993;
RX   DOI=10.1111/j.1432-1033.1983.tb07594.x;
RA   Freudl R., Cole S.T.;
RT   "Cloning and molecular characterization of the ompA gene from
RT   Salmonella typhimurium.";
RL   Eur. J. Biochem. 134:497-502(1983).
RN   [2]
RP   NUCLEOTIDE SEQUENCE [LARGE SCALE GENOMIC DNA].
RC   STRAIN=LT2 / SGSC1412 / ATCC 700720;
```

```
RX   MEDLINE=21534948; PubMed=11677609; DOI=10.1038/35101614;
RA   McClelland M., Sanderson K.E., Spieth J., Clifton S.W., Latreille P.,
RA   Courtney L., Porwollik S., Ali J., Dante M., Du F., Hou S., Layman D.,
RA   Leonard S., Nguyen C., Scott K., Holmes A., Grewal N., Mulvaney E.,
RA   Ryan E., Sun H., Florea L., Miller W., Stoneking T., Nhan M.,
RA   Waterston R., Wilson R.K.;
RT   "Complete genome sequence of Salmonella enterica serovar Typhimurium
RT   LT2.";
RL   Nature 413:852-856(2001).
CC   -!- FUNCTION: Required for the action of colicins K and L and for the
CC       stabilization of mating aggregates in conjugation. Serves as a
CC       receptor for a number of T-even like phages. Also acts as a porin
CC       with low permeability that allows slow penetration of small
CC       solutes (By similarity).
CC   -!- SUBUNIT: Monomer (Probable).
CC   -!- SUBCELLULAR LOCATION: Cell outer membrane; Multi-pass membrane
CC       protein.
CC   -!- SIMILARITY: Belongs to the OmpA family.
CC   -!- SIMILARITY: Contains 1 OmpA-like domain.
CC   -----------------------------------------------------------------------
CC   Copyrighted by the UniProt Consortium, see http://www.uniprot.org/terms
CC   Distributed under the Creative Commons Attribution-NoDerivs License
CC   -----------------------------------------------------------------------
DR   EMBL; X02006; CAA26037.1; -; Genomic_DNA.
DR   EMBL; AE006468; AAL20003.1; -; Genomic_DNA.
DR   PIR; A03436; MMEBAT.
DR   RefSeq; NP_460044.1; NC_003197.1.
DR   ProteinModelPortal; P02936; -.
DR   SMR; P02936; 22-196.
DR   PRIDE; P02936; -.
DR   GeneID; 1252588; -.
DR   GenomeReviews; AE006468_GR; STM1070.
DR   KEGG; stm:STM1070; -.
DR   PATRIC; 32380577; VBISalEnt20916_1135.
DR   eggNOG; COG2885; -.
DR   HOGENOM; HOG000274199; -.
DR   KO; K03286; -.
DR   OMA; DDNEAQK; -.
DR   ProtClustDB; PRK10808; -.
DR   BioCyc; STYP99287:STM1070-MONOMER; -.
DR   GO; GO:0009279; C:cell outer membrane; IEA:UniProtKB-SubCell.
DR   GO; GO:0005886; C:plasma membrane; IEA:UniProtKB-KW.
DR   GO; GO:0046930; C:pore complex; IEA:UniProtKB-KW.
DR   GO; GO:0015288; F:porin activity; IEA:UniProtKB-KW.
DR   GO; GO:0005198; F:structural molecule activity; IEA:InterPro.
DR   GO; GO:0000746; P:conjugation; IEA:UniProtKB-KW.
DR   GO; GO:0006811; P:ion transport; IEA:UniProtKB-KW.
DR   Gene3D; G3DSA:2.40.160.20; OMP_b-brl; 1.
DR   Gene3D; G3DSA:3.30.1330.60; OmpA/MotB_C; 1.
DR   InterPro; IPR011250; OMP_b-brl.
DR   InterPro; IPR006664; OMP_bac.
DR   InterPro; IPR002368; OmpA.
DR   InterPro; IPR006690; OMPA-like_CS.
DR   InterPro; IPR000498; OmpA-like_TM_dom.
DR   InterPro; IPR006665; OmpA/MotB_C.
DR   Pfam; PF00691; OmpA; 1.
DR   Pfam; PF01389; OmpA_membrane; 1.
DR   PRINTS; PR01021; OMPADOMAIN.
DR   PRINTS; PR01022; OUTRMMBRANEA.
DR   SUPFAM; SSF103088; OmpA/MotB_C; 1.
DR   PROSITE; PS01068; OMPA_1; 1.
DR   PROSITE; PS51123; OMPA_2; 1.
PE   3: Inferred from homology;
KW   Cell membrane; Cell outer membrane; Complete proteome; Conjugation;
KW   Disulfide bond; Ion transport; Membrane; Porin; Reference proteome;
KW   Repeat; Signal; Transmembrane; Transmembrane beta strand; Transport.
FT   SIGNAL        1     21       By similarity.
FT   CHAIN        22    350       Outer membrane protein A.
FT                                /FTId=PRO_0000020099.
FT   TRANSMEM     27     40       Beta stranded; (Potential).
FT   TRANSMEM     55     67       Beta stranded; (Potential).
FT   TRANSMEM     70     85       Beta stranded; (Potential).
FT   TRANSMEM     97    107       Beta stranded; (Potential).
FT   TRANSMEM    111    126       Beta stranded; (Potential).
FT   TRANSMEM    146    157       Beta stranded; (Potential).
FT   TRANSMEM    163    179       Beta stranded; (Potential).
FT   TRANSMEM    185    196       Beta stranded; (Potential).
FT   REPEAT      205    206       1.
FT   REPEAT      207    208       2.
FT   REPEAT      209    210       3.
FT   REPEAT      211    212       4.
FT   DOMAIN      214    342       OmpA-like.
FT   REGION      205    212       4 X 2 AA tandem repeats of A-P.
FT   DISULFID    315    327       By similarity.
FT   CONFLICT    114    114       V -> F (in Ref. 1; CAA26037).
FT   CONFLICT    247    247       S -> I (in Ref. 1; CAA26037).
SQ   SEQUENCE   350 AA;  37515 MW;  B4AC52C8C5DF54FE CRC64;
     MKKTAIAIAV ALAGFATVAQ AAPKDNTWYA GAKLGWSQYH DTGFIHNDGP THENQLGAGA
     FGGYQVNPYV GFEMGYDWLG RMPYKGDNIN GAYKAQGVQL TAKLGYPITD DLDVYTRLGG
     MVWRADTKSN VPGGPSTKDH DTGVSPVFAG GIEYAITPEI ATRLEYQWTN NIGDANTIGT
     RPDNGLLSVG VSYRFGQQEA APVVAPAPAP APEVQTKHFT LKSDVLFNFN KSTLKPEGQQ
     ALDQLYSQLS NLDPKDGSVV VLGFTDRIGS DAYNQGLSEK RAQSVVDYLI SKGIPSDKIS
     ARGMGESNPV TGNTCDNVKP RAALIDCLAP DRRVEIEVKG VKDVVTQPQA
```

Mascot: http://www.matrixscience.com/
