# Supplementary material for: Proteomic Analysis of Outer Membrane Proteins from Salmonella Enteritidis Strains with Different Sensitivity to Human Serum
Source: PLoS One. 2016 Oct 3;11(10):e0164069. doi: 10.1371/journal.pone.0164069 (PMC5047454; doi:10.1371/journal.pone.0164069)
Supplement: S6 Appendix — (DOC) [file pone.0164069.s006.doc]

A PgtE

B FliD

D OmpA

C FliD

E OmpA
